# [D-Ala^2^, D-Leu^5^] Enkephalin Attenuates Hepatic Ischemia–Reperfusion Injury in Cirrhotic Rats

**DOI:** 10.3389/fsurg.2022.839296

**Published:** 2022-05-06

**Authors:** Jueying Liu, Yuan Wang, Qianling Pan, Xueqing Chen, Yifeng Qu, Hao Zhu, Li Zheng, Yinghui Fan

**Affiliations:** Department of Anesthesiology, Ren Ji Hospital, Shanghai Jiao Tong University School of Medicine, Shanghai, China

**Keywords:** hepatic ischemia–reperfusion injury, delta opioid receptor, phosphatidylinositol-3-kinase (PI3K)/Akt pathway, cirrhotic livers, rats, DADLE

## Abstract

**Background and Aims:**

Hepatic ischemia–reperfusion injury (IRI) is a common phenomenon that occurs after liver transplantation and liver tumor surgery. It can cause liver dysfunction and recovery failure after liver surgery, even leading to acute liver failure. Our aim is to investigate the protective effect and related potential mechanism of [D-Ala^2^, D-Leu^5^] enkephalin (DADLE) treatment on hepatic IRI in cirrhotic livers of rats.

**Methods:**

The models of liver cirrhosis and hepatic IRI were established with male Sprague–Dawley rats. DADLE at a dose series of 0.5, 1, or 5 mg·kg^−1^ was injected intravenously to rats 10 min prior hepatic ischemia, followed by a 6- h reperfusion. The serum levels of alanine aminotransferase (ALT) and aspartate aminotransferase (AST), histological changes, and liver cell apoptosis were used to assess liver IRI. The optimal dose of DADLE was assessed by using the Suzuki score and ALT and AST levels. We repeated the hepatic IRI procedure on the optimal dose of the DADLE group and the delta opioid receptor (DOR) antagonist natrindole hydrochloride (NTD) injection group. Serum ALT and AST levels, histological staining, hepatic apoptosis, and serum levels of tumor necrosis factor alpha (TNF-α) and interleukin 1 β (IL-1β) were measured. The expression of protein kinase B (Akt) and its downstream proteins were evaluated by using quantitative real-time polymerase chain action (qRT-PCR) and Western blotting.

**Results:**

Compared with the control group, DADLE treatment at a dose of 5 mg·kg^−1^ reduced the Suzuki score (mean: 5.8, range: 5.0–6.6 vs. mean: 8.0, range: 7.0–8.9), the ALT level (134.3 ± 44.7 vs. 247.8 ± 104.6), and the AST (297.1 ± 112.7 vs. 660.8 ± 104.3) level. DOR antagonist NTD aggravated hepatic IRI. Compared with the control group, DADLE treatment decreased the number of apoptosis cells and microphages and neutrophils, increased the expression of Akt and its mRNA to much higher levels, and upregulated the mRNA and protein expression of Bcl-2 and Bcl-2-associated death promoter (BAD).

**Conclusion:**

DADLE treatment at a dose of 5 mg·kg^−1^ injected intravenously 10 min prior hepatic ischemia could contain rats’ hepatic IRI by activating DOR in cirrhotic livers. The effects of DADLE could be offset by NTD. The potential molecular mechanism seems to be involved in the phosphatidylinositol-3-kinase (PI3K)/Akt pathway.

## Introduction

Liver cirrhosis is the end stage of chronic liver disease, which is caused by chronic liver inflammation, followed by hepatic fibrosis. Worldwide, approximately 2 million deaths are caused by liver diseases annually, 1 million deaths are contributed by liver cirrhosis, and 1 million deaths are due to hepatitis and hepatocyte cancer (1). Liver cirrhosis is the 11th leading cause of global deaths, with high rates of worldwide morbidity and mortality (1, 2). Because patients with liver cirrhosis have poor tolerance, residual cirrhotic liver lobes would suffer severe ischemia–reperfusion injury (IRI) when patients are undergoing liver surgeries such as liver tumor resection and liver transplantation (3).

Hepatic IRI is a major cause of liver dysfunction and recovery failure after liver surgery (4), even leading to acute liver failure (5). It is caused by an interruption of blood flow during liver surgery, and then the reflowed blood carries oxygen and tissue PH, which initiates local inflammation and immune response (6). As a result, the oxidative stress and proinflammatory cytokines mediate hepatocyte necrosis and apoptosis (7).

As conventional analgesia, opioid receptor agonists have been demonstrated to reduce hepatic IRI in normal rats, including morphine (8), remifentanil (9), and sufentanil (10). Opioid receptors are G protein-coupled receptors and contain three subtypes: µ (MOR), k (KOR), and δ (DOR) opioid receptors. DOR is widely presenting throughout human body and firstly cloned in vitro in those opioid receptors (11). Unlike other opioid receptors, DOR provides beneficial effects in the form of an antidepressant, antioxidant, and neuroprotection, without unpleasant side effects such as drug abuse and respiratory depression (12). [D-Ala^2^, D-Leu^5^] enkephalin (DADLE) plays an important role in attenuating IRI in the brain (13), heart (14), intestine (15), and liver (16) by activating DOR.

The mechanism of the DADLE protective effect on the IRI of normal livers might be related to the phosphatidylinositol-3-kinase (PI3k)/protein kinase B (Akt) pathway. The PI3K/Akt pathway has been investigated to prevent cellular apoptosis and attenuate hepatic IRI (17). In normal rats, DADLE could activate the Akt pathway and modulate its downstream element phosphorylation, including glycogen synthase kinase-3b and Bcl-2-associated death promoter (BAD) (13, 18). Then, BAD regulates caspase-3 expression and inhibits hepatocytes from apoptosis. Therefore, the PI3k/Akt pathway is critical for preventing hepatic IRI, which is upregulated by DADLE treatment. However, it has not yet been reported whether DADLE triggers the PI3K/Akt pathway in cirrhotic livers. Therefore, a study of the potent mechanism of DADLE in hepatic IRI is considered very important.

In the present times, it has been found that livers in most patients undergoing liver surgery are more or less abnormal as a result of diseases such as liver cirrhosis, liver fibrosis, and liver tumor. The protective effect of DADLE has been investigated in normal livers, but there are no data on liver cirrhosis or liver fibrosis. Besides, studies on hepatic IRI worldwide have failed to address the effect of DOR on human cirrhotic livers; so this study on DOR, which is relevant to cirrhotic hepatic IRI based on the rat model, can be described as a pioneer study.

Our research focuses on finding an optimal dose, the time point, and the route of administration for DADLE; investigating the effect of DOR activation by DADLE on hepatic IRI in the livers of cirrhotic rats; studying whether the effect of DOR activation by DADLE could be canceled by NTD injected 10 min prior morphine injection; examining the PI3K/Akt pathway and its downstream proteins for studying the molecular mechanism of DADLE.

## Materials and Methods

### Ethic Statement

The present study was designed and reported according to the ARRIVE (Animal Research: Reporting of In Vivo Experiments) guidelines (19). According to the United States’ National Institutes of Health (NIH) animal care guidelines (Guide for the Care and Use of Laboratory Animals, Department of Health and Human Services, NIH Publication No. 86-23, revised 1985), all procedures were done under clean conditions and approved by the Animal Care and Use Committee of Shanghai Jiaotong University, School of Medicine (Ethic No. RJ-2017-07-10).

### Group Design

A model of cirrhotic rats was prepared according to the description of our previous study (8). Cirrhotic rats were randomly divided into two stages. In the first stage, the protective effect of DOR activation was determined. DOR agonist DADLE (Abcam, Cambridge, UK) was injected intravenously to the rats. Then, the rats were separated into five groups: SHAM, IR, and IRD (at a dose of 0.5 mg·kg^−1^), IRD (at a dose of 1 mg·kg^−1^), and IRD (at a dose of 5 mg·kg^−1^). Rats in the SHAM group received only laparotomy. Rats in the IR group were injected with saline (the same volume of DADLE) 10 min prior hepatic IRI via the left femoral vein. DADLE was diluted by saline into three concentrations. The three dosages of DADLE were injected 10 min before hepatic IRI through the left femoral vein, separately. After the pretreatment, rats were received hepatic IRI with 30-min ishchemia and a 6-h reperfusion. In the second stage, the rats were divided into four groups: SHAM, IR, IRD, and NTD groups. The dose of DADLE in the IRD group was the optimal dosage. Additionally, to detect the effect of DADLE at different reperfusion time points, the optimal protective dose of DADLE (based on the Suzuki score and serum ALT and AST levels) was used. Both IRD (the optimal protective dose of DADLE) and IR groups (only injected saline) suffered a 30-min hepatic ischemia and a 24-h reperfusion. In the second stage, to investigate the critical role of DOR in hepatic IRI, the selective DOR antagonist natrindole hydrochloride (NTD) was injected. The rats were divided into four groups: SHAM, IR, IRD, and NTD groups. The dose of DADLE in the IRD group was the optimal dosage. In the NTD (DOR antagonist natrindole hydrochloride) group, NTD (5 mg kg^−1^, R&D system, Minneapolis, MN, USA) was administered 10 min before hepatic IRI.

### Experimental Animals and Their Housing

Male Spague–Dawley rats with age ranging between 6 and 8 weeks and with a body weight of 250–300 g were obtained from the Shanghai Jiaotong University Animal Center. The rats were housed in separate cages at a room temperature of 23–25°C and at a humidity of 45%, given free access to food and water, and exposed in a standard environment with a 12- h light/dark cycle. The rats’ cirrhotic liver model was set up as described in our previous study (8). The rats were injected 99% carbon tetrachloride (CCL_4_) subcutaneously with 1:1 olive oil twice a week at a dose of 0.2 mL per 100 g of body weight for 7 weeks. Before liver IR injury, the rats’ left liver lobes were collected for histological examination to confirm the effect of CCL_4_ injection. Those who have no left liver lobes changed by CCL4 injection would be excluded from analysis.

### Experimental Procedures

#### Hepatic Ischemia–Reperfusion Injury Induction

After injecting pentobarbital 50 mg·kg^−1^ intraperitoneally, the rats’ livers were exposed through a midline laparotomy. Then, the right branch of the hepatic artery and portal vein, which supplied the right and caudate lobes, were clamped for 30 min, followed by reperfusion. After reperfusion, 2- mL blood samples were collected from the left femoral vein by using non-heparinized syringes. The caudate lobes were fixed with 4% paraformaldehyde for 48 h and embedded in paraffin. The remaining ischemic liver lobes were frozen in liquid nitrogen and transferred to a −80°C refrigerator. After removing the liver lobes, the rats were euthanized by using 50- mg·kg^−1^ pentobarbital.

### Experimental Outcomes

The primary endpoint was liver function assessed by hepatocyte injury and histological changes. Secondary endpoints were apoptosis, inflammatory cytokine expression, inflammatory cell infiltration, and Akt with downstream protein expression.

#### Evaluation of Hepatocyte Injuries

Hepatocyte injury was evaluated by measuring serum alanine aminotransferase (ALT) and aspartate aminotransferase (AST) levels. ALT (Nan-jing Jiancheng Biochemicals Ltd, China) and AST (Nan-jing Jiancheng Biochemicals Ltd, China) were detected by using an automatic procedure in the Department of Clinical Laboratory, Renji Hospital, School of Medicine, Shanghai Jiaotong University.

#### Histological and Immunohistochemical Assessment

Samples from the right lobes were collected, fixed with 4% paraformaldehyde, and cut into 4- µm slides. Then, the slides were assessed by hemotoxylin–eosin (H&E) staining. All the slides were observed under a high-power light microscope (OlympusCH30). Based on the Suzuki criteria, the histological changes were graded on a scale of 0–4; 0 denoted no necrosis, sinusoidal congestion, and vacuolation; scales between 1 and 3 denoted moderate congestion and vacuolation, and 4 denoted more than 60% necrosis. To simplify the complex scores, the sum of the Suzuki score was used as a modified parameter (20). CD11b^+^ (Abcam, Cambridge, UK) and CD68^+^ (Abcam, Cambridge, UK) antibody staining was performed according to the manufacturer’s recommendation. The percentage of positive cells was counted in 10 random fields (×400), each sample under a light microscope (Olympus CH30, Olympus, Tokyo, Japan).

#### Evaluation of Apoptosis

Transferase-mediated deoxyuridine triphosphate nick-end labeling (TUNEL; Roche Biochemicals, Mannheim, German), staining, and Western blotting of cleaved Caspase-3 expression were used for evaluating hepatocyte apoptosis. TUNEL staining was performed as described in our previous study (8). Positive brown cells were marked as apoptotic cells. The percentage of apoptotic cells was counted in 10 random fields (×400), each sample under a light microscope (Olympus CH30). The critical apoptotic protein cleaved Caspase-3 was detected by using Western blotting (see Western Blotting).

#### Evaluation of Serum TNF-α and IL-1β by Enzyme-Linked Immunosorbent Assay

The levels of serum tumor necrosis factor alpha (TNF-α) and interleukin 1 beta (IL-1β) were detected by using commercial TNF-α and IL-1β enzyme-linked immunosorbent assay kits (R&D System, Minneapolis, MN, USA). Blood samples (2 mL) were collected from the femoral vein at the end of reperfusion. Then, the blood serum was centrifuged at 3,000 g for 10 min at 4°C.

#### Quantitative Real-Time Polymerase Chain Reaction

Total RNA was extracted from the ischemic right liver lobe by using the TRIzon method (Invitrogen, CA, USA). The first strand of cDNA was synthesized by using a PrimeScript RT Reagent kit for RT-PCR (Takara, Otsu, Japan). The primers were used as follows (**Table 1**): GAPDH was the control with the following primers. All data were calculated according to the 2^−ΔΔCT^ method: ΔΔCt = (Ct Target−Ct GAPDH) test−(Ct Target – Ct GAPDH) control. Control was defined as 1.0 fold.

**Table 1:** Primer sequence for qRT-PCR.

Gene	Primer
*Akt*	Sense 5′-CTGTTGATATTCCCGTCTTTC-3′ Antisense 5′-GACTCTCCTTGCACTACATTAAC-3′
*Bcl-2*	Sense 5′-TTTGCTACAGGGTTTCATCCA-3′ Antisense 5′-CATGTTGTTGTCCAGTTCATC-3′
*BAX*	Sense 5′-TTTGCTACAGGGTTTCATCCA-3′ Antisense 5′-CATGTTGTTGTCCAGTTCATC-3′
*GAPDH*	Sense 5′- CCACAG TCCATGCCATCAC-3′ Antisense 5′- TCCACCACCCTGTT GCTGTA-3′

#### Western Blotting

Ischemic right liver lobes (30 µg) were homogenized and centrifuged at 12,000 *g* at 4°C for 15 min with lysis buffer. Proteins, whose molecular weights were above 60KD, were separated by using 10% sodium dodecyl sulfate–polyacrylamide gel (SDS–PAGE), while proteins whose weights were below 60 kD were separated by using 12% SDS–PAGE. Then, the proteins were transferred to the polyvinylidene fluoride (PVDF) membrane and blotted in 5% non-fat milk diluted by Tris-buffered saline with 0.1% Tween 20 (TBST) for 1 h. The PVDF membrane was incubated in primary antibodies for phospho-Akt (1:1,000, P-Akt; Abcam, Cambridge, UK), Akt (1:1,000, Total-Akt; Abcam, Cambridge, UK), Bcl-2 (1:1,000; Abcam, Cambridge, UK), phospho-BAD (1:1,000, P-BAD; Abcam, Cambridge, UK), BAD (1:1,000, Total-BAD; Abcam, Cambridge, UK), cleaved Caspase-3 (1:1,000; Cell Signaling Technology Inc., Danvers, MA, USA), Caspase-3 (1:1,000; Cell Signaling Technology Inc., Danvers, MA, USA), or β-actin (1:500; Epitomics, Burlingame, CA, USA) overnight at 4°C. The membranes were washed and incubated in the goat antirabbit HRP conjugate secondary antibody (1:5,000; Beyotime, Shanghai, China) for 1 h at room temperature. The signals were detected by using the enzymatic chemiluminescence plus kit. The protein band quantities were analyzed by using Image Lab (Bio-Rad Company, Hercules, CA, USA). The target proteins were normalized to the level of β-actin in the same sample.

### Statistical Analysis

All data were expressed as mean ± SD (standard deviation). Except for the H&E staining scores, it was reported as median and ranges. Analysis was performed by using Graphpad 9.0 for Mac (San Diego, CA, USA). One-way analysis of variance (ANOVA), followed by Turkey’s test, was used for making multiple comparisons. For the H&E staining scores, nonparametric one-way ANOVA, followed by the Kruskal–Wallis test, was used among these multiple groups. A value of *P* < 0.05 was considered statistically significant. The sample size was examined by using G′ Power software 3.1.7 version (Franz Faul, Universität Kiel, Germany).

## Results

### Inclusion of Animals

Totally, 164 rats were involved in these studies. After CCL_4_ injection of rats, 42 rats were excluded, as 14 of them died and 28 rats had negative biopsy results. The remaining 122 rats completed the study, and each group had at least eight rats.

### Outcomes and Estimations

#### The Effect of [D-Ala^2^, D-Leu^5^] Enkephalin on Hepatic Ischemia–Reperfusion Injury in Cirrhosis

**Figure 1:**
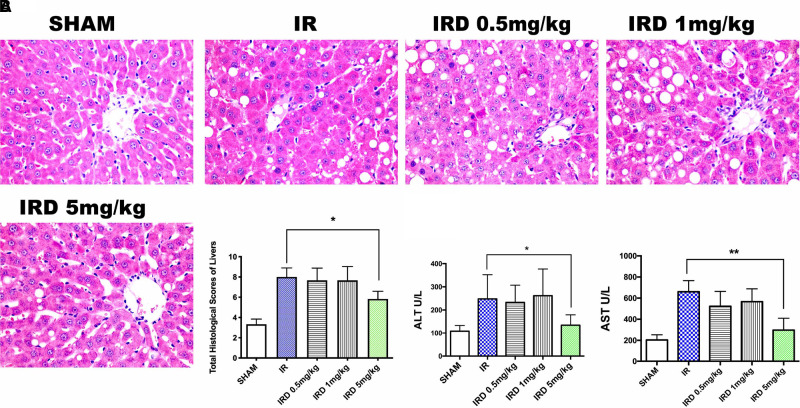
DADLE treatment attenuated hepatic IRI in cirrhotic livers by decreasing pathological scores and ALT and AST levels. (**A**) H&E staining and Suzuki score after a 6- h reperfusion. Original magnification 400×, *n* = 6, *p < 0.05. (**B**) Serum ALT and AST levels in each group after 6 h reperfusion; *n* = 6, *p < 0.05; **p < 0.01. IR, ischemia–reperfusion injury group; IRD, DOR agonist DADLE treatment group.

First of all, in order to investigate the effect of DOR activation on hepatic IRI, the histological changes were used. Compared with the IR group, DOR agonist DADLE at 5 mg·kg^−1^ could significantly contain liver structure damage, areas of necrosis, and rapid degeneration, according to the total Suzuki scores (IRD 5 mg·kg^−1^ vs. IR; mean: 5.8, range: 5.0–6.6 vs. mean: 8.0, range: 7.0–8.9; *p *= 0.0006) (**Figure 1A**). After 24 h of reperfusion, there were no differences among the SHAM, IR, and IRD (5 mg·kg^−1^) groups.

The serum enzymes were also used for evaluating hepatic IRI. The serum levels of ALT and AST significantly decreased in the DADLE treatment (IRD, 5 mg·kg^−1^) group (ALT: IRD vs. IR; 134.3 ± 44.7 vs. 247.8 ± 104.6, *p *= 0.004; AST: IRD vs. IR; 297.1 ± 112.7 vs. 660.8 ± 104.3, *p *< 0.0001, respectively) (**Figure 1B**). However, the dose dependence of DOR activation was not observed because there were no differences among the IRD groups. No significant differences were found in those groups even 24 h after reperfusion.

#### Selective Delta Opioid Receptor Antagonist Eliminates the Beneficial Effect of [D-Ala^2^, D-Leu^5^] Enkephalin

**Figure 2:**
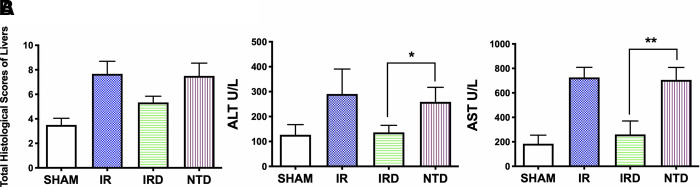
DOR antagonist NTD eliminated the beneficial effect of DADLE. (**A**) Suzuki score after a 6- h reperfusion by H&E staining. *n* = 6, *p < 0.05 compared with the IR group. (**B**) Serum ALT and AST levels in each group after 6- h reperfusion; *n* = 6, *p < 0.05; **p < 0.01. IR, ischemia–reperfusion injury group; IRD, DOR agonist DADLE at 5 mg·kg^−1^ treatment group; NTD, DOR antagonist natrindole hydrochloride administered group.

Secondly, to demonstrate the critical role of DOR in hepatic IRI, the selective DOR antagonist NTD was injected. Although there was no significant differences, NTD increased the total Suzuki scores after a 6- h reperfusion (IRD 5 mg·kg^−1^ vs. NTD; mean: 5.6, range: 4.8–6.5 vs. mean: 7.5, range: 6.3–8.6; *p *= 0.2582) (**Figure 2A**). Compared with the IRD group, the serum levels of ALT and AST were elevated by NTD injection (ALT: IRD vs. NTD; 136.3 ± 28.5 vs. 259.0 ± 58.2, *p *= 0.0029; AST: IRD vs. NTD; 260.8.1 ± 109.3 vs. 706.5 ± 101.8, *p *< 0.0001). These indicated the importance of DOR in hepatic IRI (**Figure 2B**).

#### [D-Ala^2^, D-Leu^5^] Enkephalin Treatment Reduces Apoptosis While Eliminating the Delta Opioid Receptor Antagonist

**Figure 3:**
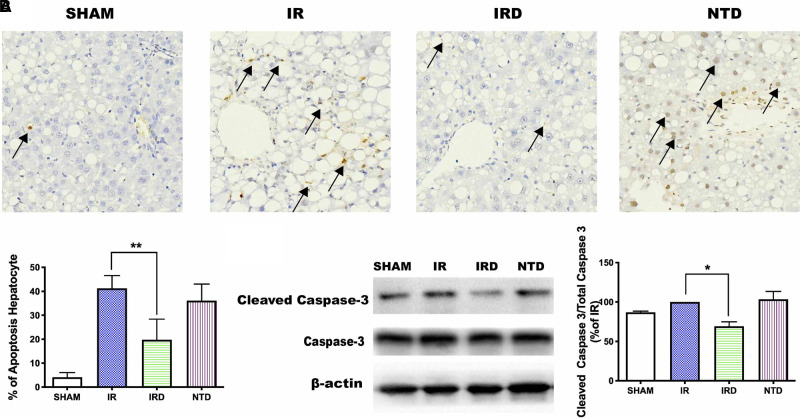
DOR antagonist NTD eliminated the beneficial effect of DADLE. (**A**) TUNEL staining after 6 h reperfusion. Original magnification 400×, *n* = 6, *p < 0.05 compared with the IR group, **p < 0.01 compared with the IR group. (**B**) The expression of cleaved Caspase-3 levels in each group after 6 h reperfusion; *n* = 6, *p < 0.05 compared with the IR group. IR, IRD, and NTD are the corresponding rat groups as in Figure 2. The black arrow indicates the apoptotic liver cell.

Thirdly, to evaluate the effect of DOR activation on hepatocyte apoptosis, the positive cell numbers were counted by TUNEL staining and the Cleaved Caspase-3 expression was detected by western blotting. Compared with the IR group, DOR activation could decrease the ratio of apoptotic cell numbers (IRD vs. IR: 19 ± 8.5% vs. 41 ± 5%, *p *< 0.0001; **Figure 3A**) and downregulate Cleaved Caspase-3 expression (IRD vs. IR: 69.1 ± 5.7% vs. 100%, *p *< 0.0001; **Figure 3B**). On the other hand, DOR antagonist NTD could increase the number of positive cells and the expression of Cleaved Caspase-3.

#### [D-Ala^2^, D-Leu^5^] Enkephalin Treatment Reduces Immune Responses in Cirrhotic Hepatic Ischemia–Reperfusion Injury, Which Was Reversed by Delta Opioid Receptor Antagonist Natrindole Hydrochloride

**Figure 4:**
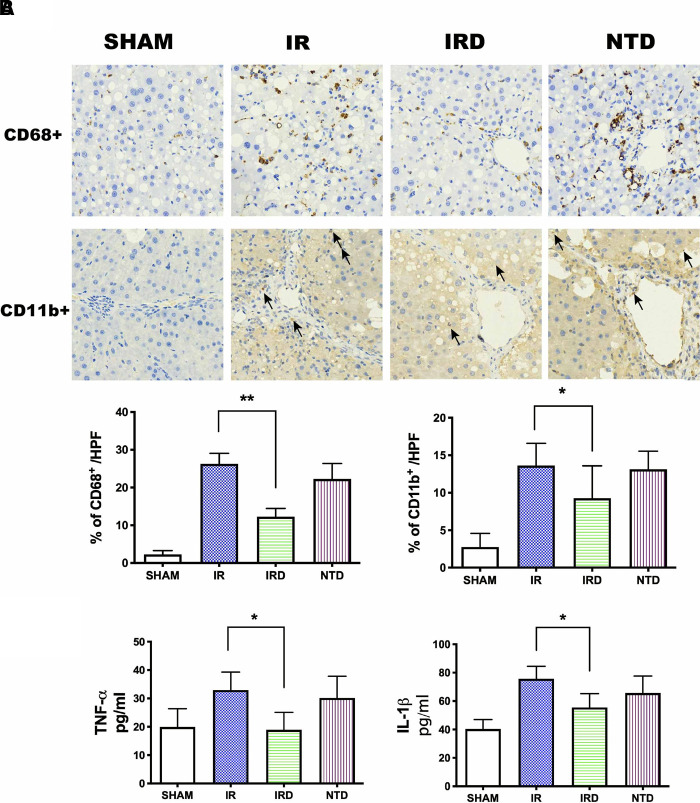
DADLE treatment reduced inflammatory cell infiltration and serum cytokines levels. (**A**) Immunohistochemistry staining for and after 6 h of reperfusion. Original magnification 400×, *n* = 6, *p < 0.05 compared with the IR group, **p < 0.01 compared with the IR group. The CD68^+^ positive cells were presented as brown. The black arrow indicates the CD11b^+^ positive cell. (**B**) Serum concentrations of TNF-α and IL-1β were lower in the IRD groups compared with the IR group; *n* = 6, *p < 0.05 compared with the IR group. IR, IRD, and NTD are the same rat groups as in Figure 2.

Fourthly, we examined if DADLE treatment could result in decreasing microphage (CD68^+^) and neutrophil (CD11b^+^) infiltration, destroying proinflammatory cytokine (TNF-α and IL-1β) levels as well. DADEL treatment could significantly decrease the ratio of CD68^+^ and CD11b^+^ numbers compared with the IR groups, respectively (IRD vs. IR; 12.3 ± 2.19% vs. 26.3 ± 2.8%, *p *< 0.01; 11.5 ± 4.04% vs. 17.3 ± 5.2%, *p *< 0.05; **Figure 4A**). Meanwhile, DADLE treatment could also lower the expression of TNF-α (IRD vs. IR; 19.0 ± 6.2 vs. 33.0 ± 6.3, *p *= 0.003) and IL-1β (IRD vs. IR; 55.5 ± 9.7 vs. 75.7 ± 8.8, *p *= 0.001) (**Figure 4B**). However, these beneficial effects were eliminated by DOR antagonist NTD (**Figure 4A, B**).

#### The mRNA Expression and Protein Phosphorylation of PI3K/Akt Pathway Induced by [D-Ala^2^, D-Leu^5^] Enkephalin Treatment

**Figure 5:**
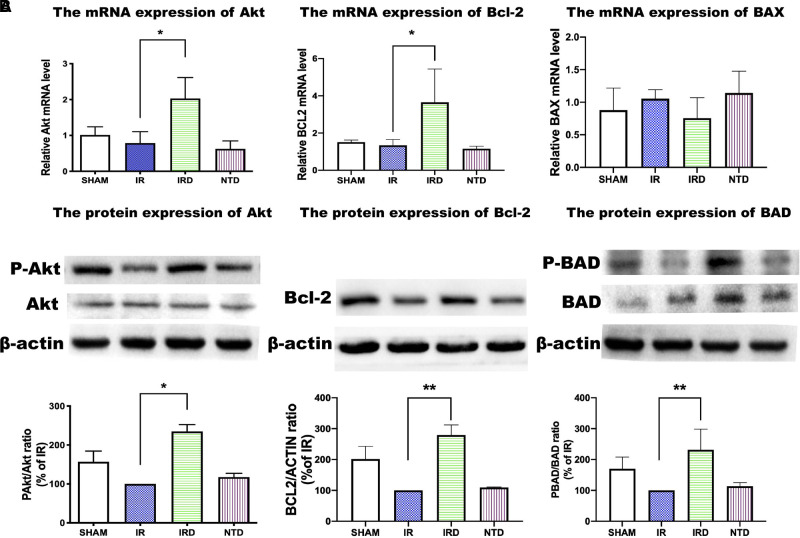
The mRNA and protein expression of the PI3K/Akt pathway in each group. (**A**) mRNA of Akt, Bcl_2_, and BAX measured by qRT-PCR. *n* = 6, *p < 0.05 compared with the IR group. (**B**) DADLE upregulated the expression of Akt, Bcl-2, BAD, compared with the IR group; *n* = 6, *p < 0.05 compared with the IR group, ** *p* < 0.01 compared with the IR group. IR, IRD, and NTD mean the same as in Figure 2.

In our study, we found that the mRNA of Akt and Bcl-2 was increased in the DADLE treatment group (IRD vs. NTD; 2.03 ± 0.58 vs. 0.79 ± 0.32, *p *= 0.0136) (**Figure 5A**). Although the expression of Bcl-2-associated X (BAX) had no significant differences between the IRD and the IR groups, it showed decreasing trends in the DADLE group (**Figure 5A**).

Besides, we found that the phosphorylated protein levels of the prosurvival kinase Akt were elevated in the DADLE treatment group (IRD vs. IR; 234.8 ± 17.4 vs. 100, *p *< 0.0001). The effect of DADLE was completely eliminated by DOR antagonist NTD (**Figure 5B**). Bcl-2 and BAD, the downstream protein regulated by Akt, were evaluated by using Western blotting. In this research, the level of phosphorylated Bcl-2 increased after DOR activation compared with that in the IR group (IRD vs. IR; 279.2 ± 33.0 vs. 100, *p *< 0.0001). Also, the expression of BAD was higher in the IRD group than in the IR group (IRD vs. IR; 231.7 ± 66.8 vs. 100, *p *= 0.002) (**Figure 5B**). These effects could be offset by DOR antagonist NTD treatment (**Figure 5B**).

## Discussion

Since the delta opioid receptor (DOR) was discovered by scientists, its role was investigated in neurological diseases and pain control fields (11). It was reported that the IRI in the heart (21), spinal cord (22), and brain (23) could be alleviated and neurological recovery promoted when DOR was activated by DADLE. Like other organs, the liver also contains many DORs (24). In a study of hepatic IRI in normal rats, DADLE could protect hepatocytes by reducing the cellular metabolism (25). Besides, it was observed that DADLE could prevent cellular mitochondrial dysfunction through opioid receptor signaling involvement in vitro (16). In this paper, the beneficial effects of DOR activation and potential mechanism were further studied and identified in the hepatic IRI model in cirrhotic rats: (1) The optimal protective dose of DADLE in hepatic IRI (5 mg·kg^−1^), the administration route, intravenous injection, and the time point, 10 min prior hepatic ischemia, followed by a 6- h reperfusion, were identified. (2) The protective effects could be eliminated by DOR-selected antagonist NTD. (3) DOR activation by DADLE could not only reduce hepatocytes’ apoptosis, but also prevent cellular inflammation. (4) The molecular mechanism of DOR activation might be related to the upregulated PI3K/Akt pathway.

The rational methods to evaluate hepatic IRI in cirrhotic rats are based on serum ALT and AST levels and histological changes through Suzuki scores (6). But, we found that it was difficult to evaluate the protective effect of DADLE by histological staining, because the livers were separated into several areas by the proliferate vascular and bile ducts after CCL_4_ induced liver cirrhosis. There were no clear differences between the control group and the treatment group. The reason was that the preinjury and structural damage caused by CCl_4_ made the histological appearance much more complex (26). Therefore, we used the modified Suzuki scores, and found that these were better at evaluating visual hepatic injury. We clearly observed that DADLE could reduce the serum ALT and AST levels, vacuolation, necrotic areas, and cellular infiltration through histological score assessment. We chose 6- h reperfusion due to the peak levels of aminotransferases in the serum ranging between 4 and 8  after reperfusion (27). Compared with the 6- h reperfusion, we found that there were no differences in the serum levels of ALT and AST among the SHAM, IR, and IRD groups after 24 h of reperfusion. This indicated that the time point for the protective effect of DADLE is at the 6- h reperfusion point. In our research, the optimal pretreatment dosage was also at 5 mg·kg^−1^, but it was injected intravenously. We also found that DADLE pretreatment had no dose-dependent response. Our cirrhotic liver model had a widely branched vascular network and DADLE probably could not reach all the lobes equally if it was administered intravenously.

In our study, DOR antagonist NTD was used to determine the role of DOR in hepatic IRI protection. The selected DOR antagonist NTD could totally suppress the beneficial effect of DADLE. These indicated the crucial role of the DOR-dependent mechanism in DADLE hepatoprotection. To our knowledge, the question whether opioid receptors have a role to play in the hepatic IRI protective effect is controversial. Liu et al. observed that naloxone could not totally suppress the beneficial effects of MOR agonist remifentanil. This indicated that remifentanil mimics the OR independent mechanism on hepatic protection (28). These findings were not surprising, because remifentanil is not only an active opioid receptor, but also triggers a toll-like receptor 4 (29). Therefore, remifentanil could not be totally inhibited by naloxone. The main difference lies in the presence of different opioid receptor agonists.

A study on the mechanism of DOR activation in hepatic IRI was also initiated. Recently, other researchers found that DOR triggered a protective effect by reducing cellular metabolism, which resulted in ameliorating hepatic IRI (30). It was also found that DOR activation by DADLE could preserve perfused liver lobes via the antiapoptosis pathway (16). Apoptosis is a major element for cellular death after hepatic IRI. Caspase-3 plays a crucial role in mediating the apoptosis procedure (31). PI3K/Akt has been demonstrated to have beneficial effects on cellular survival and suppressing apoptosis (32). Once Akt was activated, its downstream proteins BAD and Bcl-2 were phosphorylated, and then the expression of BAX and capase-3 was inhibited. In this study, we found that DADLE treatment could increase the mRNA and protein expression levels of Akt, BAD, and Bcl-2 and decrease the expression of Cleaved Capsase-3 and BAX. But, the expression of these proteins could be preserved by NTD. These findings supported that DADLE treatment inhibited cellular apoptosis via the PI3K/Akt pathway.

We also found that DADLE treatment could decrease the levels of proinflammatory cytokines TNF-α and IL-1β. TNF-α and IL-1β are macrophage-derived and neutrophil-derived cytokines, respectively (33). In our work, we found that the ratio of macrophage (CD68^+^) and neutrophil (CD11b^+^) numbers could be significantly decreased by DADLE treatment. This means that DADLE treatment can prevent macrophage and neutrophil cell infiltration. On the contrary, cytokines, macrophage, and neutrophil cells were increased by the DOR antagonist, NTD. A study of the molecular pathway to examine the anti-inflammatory effect of DADLE will be our next research endeavor.

The key role of the PI3K/Akt pathway in the DADEL protection effect remains unexplained in this work. Possibly, we will use the Akt inhibitor in a future investigation. Secondly, this was an animal study, and, therefore, we could not predict the response in humans. Thus, further clinical studies are awaited.

In summary, our results demonstrated the DOR agonist DADLE at an optimal protective dose, 5 mg·kg^−1^, injected intravenously, and 10- min prior hepatic ischemia could attenuate hepatic IRI in cirrhotic livers by DOR activation. The protective effects of DADLE could be eliminated by the DOR-selected antagonist NTD. DOR activation by DADLE could not only reduce hepatocytes’ apoptosis, but also prevent cellular inflammation. The molecular mechanism of the protective effects of DADLE on hepatic IRI could be related to the upregulated PI3K/Akt pathway. These conclusions may provide new insights into opioid receptor hepatocyte protection in cirrhotic livers, which could be a potential molecular target for therapeutic methods. The detailed molecular mechanism of DADLE in hepatic IRI and its use in the clinic is reserved for our future studies.

## Data Availability

The raw data supporting the conclusions of this article will be made available by the authors without undue reservation.
